# The Dental Colleges

**Published:** 1896-01

**Authors:** 


### EDITORIAL.

### The Dental Colleges.

In every effort to elevate the standard of dental education by increased requirements for entrance and graduation and for an extension of time, as a requirement for graduation the objection has almost invariably been raised that it would cut down the number in attendance, and to such a degree as to cripple some, if not many, of our colleges. But what are the facts? In nearly all the colleges there is an increase in the number of students each year during the period that these advances have been made, and now that the requirements are higher than in the past, there is, in the aggregate, a much larger number of students than ever before. While the unfit are being rejected or kept away a better class of students are being attracted to the colleges, and they greatly appreciate the advancement that has been made, and the better work that necessarily results under the improved adjustment of curriculæ, or schemes of study and work.

While this advance was being urged by some there were others, and some of the best men in the profession, who earnestly opposed it as being impracticable and very likely disastrous to the colleges; but when the point was carried they yielded to the will of the majority, and with one accord all the members of the Faculties Association united in the experiment, as it was called by some. And now it would be difficult to find any who would confess that they ever were opposed to it, and all now are in full harmony with the advances that have been made and are endeavoring to comply fully with the new requirements.

The schools have in no sense been injured, but, on the other hand, made better in all respects by the advances inaugurated.

While great benefit will accrue to all concerned by the progress that has been made, it ought to be borne in mind that further advance will be demanded in the not distant future; perfection is not, by any means, yet attained, and it is not possible to set, or even suggest, a limit to the advance of the future.

The man imbued with the spirit of the age will not rest content with defective methods nor imperfect results.

The means by which greater advance may be made ought to be a subject of study by every educator.

